# Mitigating wildfire carbon loss in managed northern peatlands through restoration

**DOI:** 10.1038/srep28498

**Published:** 2016-06-27

**Authors:** Gustaf Granath, Paul A. Moore, Maxwell C. Lukenbach, James M. Waddington

**Affiliations:** 1 Department of Ecology, Department of Aquatic Sciences and Assessment, Swedish University of Agricultural Sciences, Box 7044, 75007 Uppsala, Sweden; 2 School of Geography and Earth Sciences, McMaster University, Hamilton, ON, L8S 4K1, Canada

## Abstract

Northern peatlands can emit large amounts of carbon and harmful smoke pollution during a wildfire. Of particular concern are drained and mined peatlands, where management practices destabilize an array of ecohydrological feedbacks, moss traits and peat properties that moderate water and carbon losses in natural peatlands. Our results demonstrate that drained and mined peatlands in Canada and northern Europe can experience catastrophic deep burns (>200 t C ha^−1^ emitted) under current weather conditions. Furthermore, climate change will cause greater water losses in these peatlands and subject even deeper peat layers to wildfire combustion. However, the rewetting of drained peatlands and the restoration of mined peatlands can effectively lower the risk of these deep burns, especially if a new peat moss layer successfully establishes and raises peat moisture content. We argue that restoration efforts are a necessary measure to mitigate the risk of carbon loss in managed peatlands under climate change.

Throughout the Holocene, northern peatlands have been persistent terrestrial carbon sinks that are estimated to contain approximately one-third of global soil carbon^1^. Numerous negative ecohydrological feedbacks maintain a high water table (WT)^2^ and an abundance of peat mosses (genus *Sphagnum*) with ecophysiological traits that enable long-term accumulation of peat by limiting decomposition^3^,^4^ and wildfire combustion^5^. Wildfire is the largest disturbance affecting northern peatlands, accounting for >97% of all disturbances (by area) and accounting for annual carbon losses of 0.005 Gt in western Canada alone^6^. These peat fires have (shallow) burn depths of 1–10 cm, typically consuming 2–3 kg C m^−2 ^5,7, which is often re-sequestered in 60–140 years post-fire^8^,^9^,^10^. Given that fire return intervals can be as short as 100–150 years in sub-humid continental peatlands^9^, and may exceed 2000 years in humid climates^11^, northern peatlands are generally resilient to wildfire^12^. In contrast, tropical/temperate peatlands drained for forestry/agricultural operations can have deep burn depths of 40–50 cm and release an average of 30–45 kg C m^−2 ^13,14. For example, wildfires in drained Indonesian peatlands have annual carbon emissions often exceeding 0.25 Gt^15^, where emissions during the 1997 El Niño event reached a massive 0.95–2.57 Gt^13^,^15^, equivalent, at that time, to 13–40% of global fossil fuel emissions^13^. These peat fires are a major environmental problem and also contribute to large declines in biodiversity and ecosystem functioning^16^. Moreover, impacts on human health are an acute problem, as peat smoke pollution causes immediate and delayed effects on mortality^17^.

The high burn severity of drained tropical/temperate peatland fires suggests that large-scale peatland drainage and mining in northern peatlands over the last century has also likely made managed northern peatlands more vulnerable to wildfire than natural (undrained) peatlands. Recent research showed that an Alberta peatland drained for forestry and an Ontario peatland drained and mined for agriculture and horticulture experienced wildfire carbon losses of 16.8 kg C m^−2 ^10 and 15.5 kg C m^−2 ^18, respectively. Moreover, the large negative consequences of high burn severity in managed northern peatlands was exemplified in 2010 when Russia was affected by several hundred boreal peat fires. Intense smouldering in drained and mined peatlands not only made fire suppression difficult and costly but also, like in Indonesia, caused smoke pollution that has been linked to increased human mortality (over 3,000 deaths) in Moscow^19^. Future warmer and drier conditions as a direct consequence of climate change, will likely result in an increase in boreal wildfire frequency, severity and area burned^20^ and an associated increase in northern peatland fire severity^21^. With 21 Mha of northern peatlands having been mined or drained for forestry (>90% in European Russia and Fennoscandia alone^22^, Supplementary Table 1), it is especially pressing to develop mitigation strategies to reduce the risk of deep burning in these managed ecosystems^23^. While peatland rewetting and restoration have been suggested as useful management strategies for lowering the risk of deep burns in northern peatlands^24^, evidence-based tools to evaluate the risk of severe peat fires and the effectiveness of mitigation strategies are lacking. In order to address this need, the objectives of this study are to: (i) determine the historical and future risk of managed northern peatlands to wildfire and (ii) assess how management strategies (peatlands restoration and rewetting) can be used to potentially mitigate deep burning in these peatlands.

To achieve our goals, we compare historical and future water availability (maximum potential water deficit) in managed peatlands and synthesize the current knowledge on the causes of contemporary deep burning. We collected data (new and literature values) on bulk density (*ρ*_*b*_) profiles and mean seasonal WT position data in natural, drained, mined and restored peatlands to model WT position under various peatland management scenarios (natural, drained, mined, and restored) for both historical and future water deficit scenarios. Water table data and *ρ*_*b*_ data were then used to parameterize the Peat Smouldering and Ignition (PSI) model^25^,^26^ to compare the peat smouldering potential (risk of deep burning) of various managed peatland scenarios. Our study focus is on both mined and peatlands drained for forestry, and does not cover peatlands drained for farming or grazing.

## Results

### Historical and future managed peatland water table position

**Figure 1:**
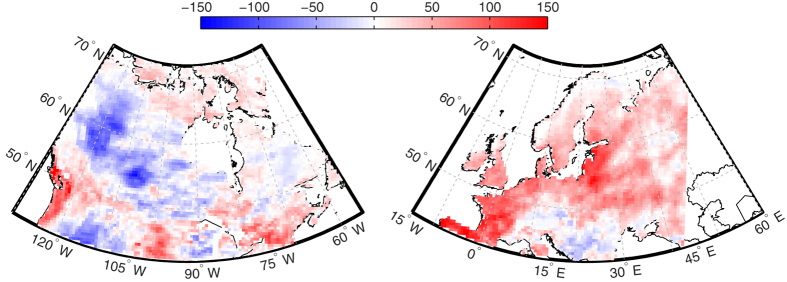
Absolute difference of average annual maximum water deficit, in mm, for North America (left) and Europe (right) between the periods 1981–2000 and 2041–2060 for the RCP 8.5 emission scenario generated from Canadian Regional Climate Model (CRCM 4, Canadian Centre for Climate Modelling and Analysis, Environment Canada; URL: http://www.cccma.ec.gc.ca/data/canrcm/CanRCM4/index_cordex.shtml; accessed: Oct.16, 2015). Red colour indicates less water available in 2041–2060. Resolution 0.44 × 0.44 grid spacing. Map was generated using Matlab R2010a v.7.1. software.

To model changes in WT position due to climate change, we investigated the temporal and spatial variation on maximal potential water deficit. Our results show a general future increase in maximum potential water deficit, i.e. less water available, of 40 ± 25 mm (mean ± sd) across Fennoscandia, the Baltic countries, European Russia, Poland, and Belarus (Fig. 1), where the majority of drained peatlands in the northern hemisphere are located (Supplementary Table 1). For North America, an increase in water deficit is largely confined to the temperate and tundra zone, while much of the western boreal zone is predicted to experience a decrease in maximum potential water deficit.

**Figure 2:**
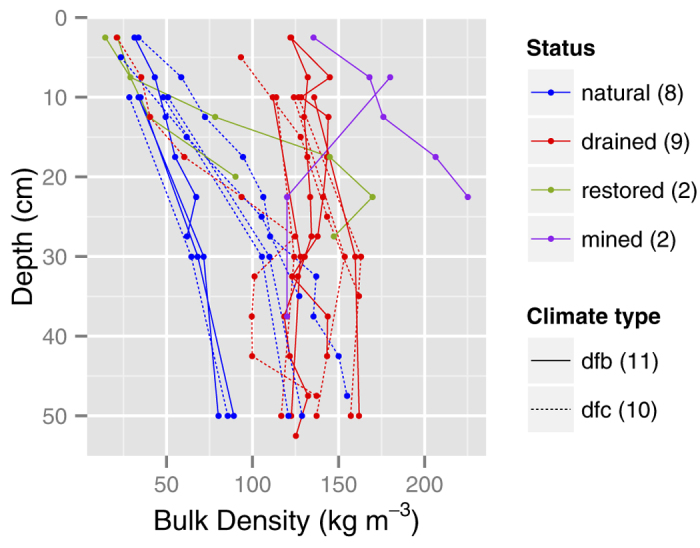
Mean bulk density (*ρ*_*b*_) profiles for five areas in Finland and five peatlands in Canada. Natural and drained profiles were obtained from each peatland except for two peatlands in Canada where only a drained profile is included. The mined and the restored peat profiles furthest to the right below 20 cm are from the same site. The two climate types (Dfb = warm summer continental or hemiboreal, Dfc = continental subarctic or boreal) are according to the Köoppen-Geiger climate type.

Bulk density (*ρ*_*b*_) is a key peat property that affects peat moisture retention and WT response to water deficit. We found a clear difference in *ρ*_*b*_ between drained and natural peatlands in both North America and Northern Europe (Fig. 2). On average, the drained peatlands have a *ρ*_*b*_ near 125 kg m^−3^ throughout the top peat layer (0–60 cm). The natural peatlands have a much lower *ρ*_*b*_ near the surface (25 kg m^−3^), which increases with depth and may reach 125 kg m^−3^ 40 cm below the surface. However, such high *ρ*_*b*_ at 40 cm are associated with a boreal-subarctic continental climate. The mined peatland profile has a surface *ρ*_*b*_ of 125 kg m^−3^, while the restored peatland has a surface *ρ*_*b*_ similar to natural peatlands. The natural peatland profiles retrieved from a database^27^ were generally within the range of natural peatlands from our paired studies. Only one site, a coastal peatland, had *ρ*_*b*_ above 100 kg m^−3^ in the top 0–20 cm.

**Figure 3:**
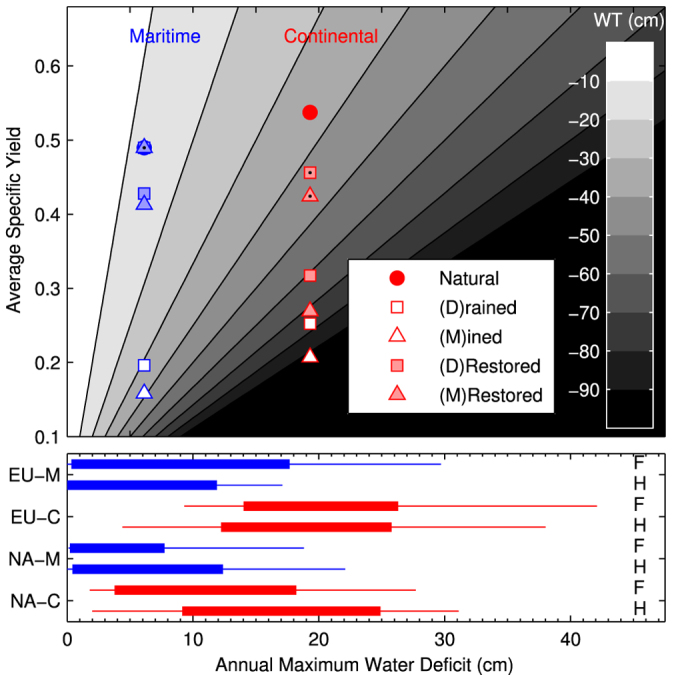
Lower panel shows the distribution of maximum cumulative water deficit for continental (C, red) and maritime (M, blue) climate locations in North America (NA) (NA-M Saint Charles de Bellechasse, QC; NA-C Athabasca, AB) and Europe (EU) (EU-C Moscow, RU; EU-M Fajemyr, SE) under an historical (H: 1981–2000) and future (F: 2041–2060) climate change scenario (CRCM4, RCP8.5). Upper panel shows the simulated effect of peatland management on maximum water table (WT) drawdown based on average water deficit and idealized specific yield profiles for a maritime and continental bog with an average spring water table position at the surface. Specific yield (*S*_*y*_) is the depth of water that must be added to or removed from a soil column in order to cause a unit rise or fall in the WT. Specific yield is directly linked to peat bulk density (*ρ*_*b*_) through pore space and moisture retention characteristics. Restored points with and without a black dot correspond to a restored *Sphagnum*/peat layer of 28 and 12 cm, respectively (maritime 28 cm restored omitted for clarity). The grayscale represents the change in WT position and the distance between points within the same climate zone illustrate the effect of the management practices on WT change.

Using *ρ*_*b*_ profiles idealized from Fig. 2 and a simple water balance model (see Methods), the broad effects of regional climate and peatland management on WT drawdown are shown to have strong interactive effects, while the effect of climate change for the emission scenario and time-frame presented is of secondary importance (Fig. 3). While drained peatlands have a lower ‘initial’ WT position, our modeling exercise demonstrates that drained peatlands undergo an approximately two times greater maximum WT decline compared to natural peatlands given the same water deficit (Fig. 3) and initial conditions. The maximum WT decline for a mined peatland is three times greater than the decline of natural peatlands. However, the effect of drainage and mining on the absolute WT drop is smaller compared to the effect of climate region. For example, the natural, drained and mined scenario WT declines were 13, 32 and 40 cm, respectively, for a maritime climate but increased to 36, 77 and 94 cm, respectively, for the continental climate (Fig. 3). Our modelled restoration scenarios suggest that a 12 cm layer of growing *Sphagnum* is sufficient to remove the large WT decline in drained and mined peatlands located in a maritime climate, as the maximum water decline is only 9 cm. However, in a continental climate, a thicker *Sphagnum* moss layer is required to achieve the same effect, as evidenced from our 28 cm moss layer results. A restored moss layer of only 12 cm in a continental climate peatland reduces the WT decline by 16 and 22 cm for drained and mined peatlands, respectively.

The effect of a future increase in maximum water deficit (see Fig. 1) on WT can be assessed by shifting to the right along the x-axis in Fig. 3. For example, the modelled mean increase in annual water deficit of 50 mm for the European maritime climate location (Fig. 3, lower panel) may result in a further WT decline of approximately 20 cm for drained and mined peatlands but only a 10 cm decline in natural peatlands.

### Burn severity modelling

**Figure 4:**
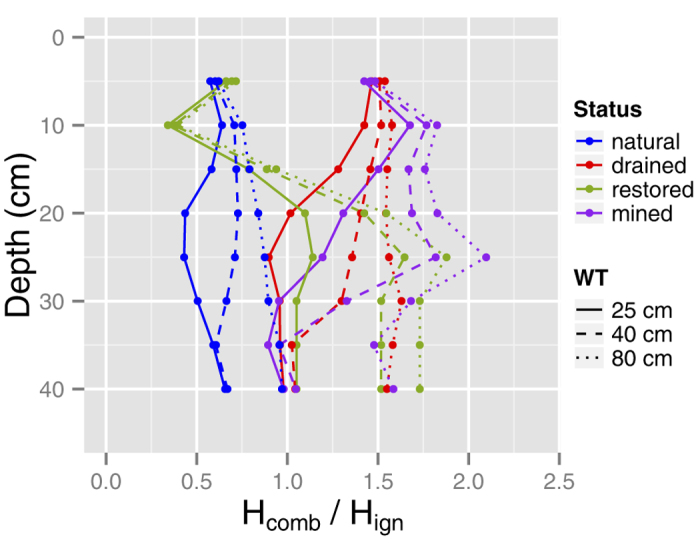
Combustion risk, expressed as the quotient between energy release (*H*_*comb*_) and energy required for ignition (*H*_*ign*_) between successive 5 cm peat layers, for various peatland types at water table (WT) depths of: (i) 40 cm, which is a typical WT in drained peatlands; (ii) 80 cm, which is a more extreme scenario supported by the difference in WT decline in drained peatlands between wetter and drier climatic zones; and (iii) 25 cm, which represents a rewetting scenario close to natural conditions. An *H*_*comb*_/*H*_*ign*_ quotient <1 indicates that the combustion risk is zero (i.e. downward smouldering propagation ceases), but a more realistic cut-off is between 1–1.5.

The PSI model relies on *ρ*_*b*_ and gravimetric water content to predict the risk of smouldering propagation between the superjacent (*H*_*combustion*_) and the subjacent (*H*_*ignition*_) peat layer (*H*_*comb*_/*H*_*ign*_ quotient, see Fig. 4 and Methods for details). Based on the *ρ*_*b*_ data (Fig. 2), we prescribed surface *ρ*_*b*_ values of 25, 25, 100 and 125 kg m^−3^ for natural, restored, drained and mined peatlands, respectively. Results demonstrate how the risk varies with peatland management and height above the WT (Fig. 4). Deep burning of up to 30 cm is possible for a WT depth of 40 cm in drained and mined peatlands and this decreases to ~20 cm for the rewetting scenario (WT = 25 cm). Much deeper burns are possible at a WT depth of 80 cm in both drained and mined peatlands. In contrast, the *H*_*comb*_/*H*_*ign*_ quotient is <1 for the upper 15 cm in both restored and natural peatlands, which means that insufficient energy is released upon combustion to sustain downward smouldering. To test if our *H*_*comb*_/*H*_*ign*_ modeling approach provides probable predictions, we ran our models for two managed peatlands that burned in Canada: (i) Salteaux, AB, drained for forestry (mean depth of burn, DOB = 21 cm)^10^; and (ii) Wainfleet, ON, abandoned mined peatland (mean DOB = 24 cm)^18^. Model predictions were in line with the actual risk of deep burning in the drained/mined peatland (Supplementary Fig. 1), suggesting our models are providing realistic risk assessments.

## Discussion

### Peatland drainage and mining enhances peat burn severity

Wildfire activity in the boreal is expected to increase in the near future in part due to the drying of previously wet peatlands as a direct consequence of climate change^20^. Our water deficit modelling supports this research and demonstrates that there is a clear risk of greater water deficits in northern Europe, where large portions of drained peatlands are located (i.e. European Russia and Fennoscandia, Fig. 1, Supplementary Table 1). Our results indicate that increasing water deficits will result in greater WT drawdowns, and even greater burn severity, in drained and mined peatlands. As such, land-use change and climate change are both acting to enhance northern peatland wildfire risk. We attribute this increased risk to a loss of negative ecohydrological feedbacks and peat mosses (*Sphagnum*) that function to moderate WT drawdowns during drought^2^.

**Figure 5:**
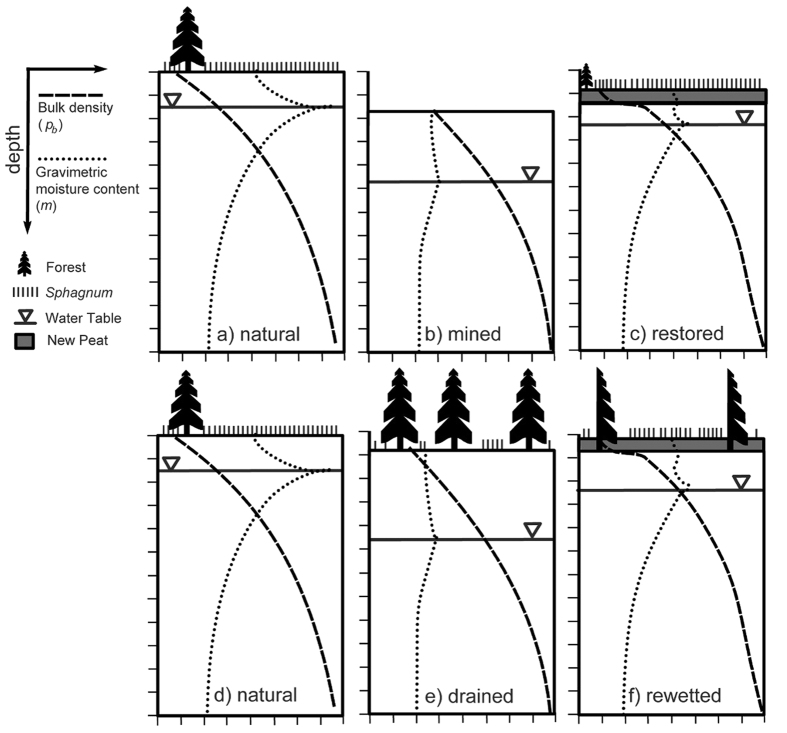
The effect of drainage, mining, rewetting and restoration on relative bulk density and gravimetric water content in peatlands. (**a**–**c**) Illustrate the impact of mining (drainage with the top peat layer removed) on a natural peatland and the response of restoration (rewetting and the establishment of a *Sphagnum* moss layer). (**d**–**f**) Illustrate the impact of drainage for forestry on a natural peatland and the response of tree removal and rewetting. These scenarios do not include non-*Sphagnum* dominated peatlands such as rich fens but see text for discussion.

We have synthesized our results into a conceptual model to illustrate the interaction between peatland management, peat/moss hydrophysical properties, and ecohydrological feedbacks in regulating peatland burn severity (Fig. 5). Natural peatlands are characterized by a high WT, high gravimetric moisture content (*m*) and low surface bulk density (*ρ*_*b*_) (Fig. 5a,d). These wet conditions are mainly maintained by the high abundance of peat mosses through several key traits (e.g. water storage in hyaline cells, external water transportation, low decay rate^4^). Previous research has shown that boreal peat can smoulder at *m* levels up to 295%^25^. However, *m* levels are seldom this low in natural peatlands that have a high abundance of peat mosses (Fig. 5a), making the H*_ign_* term in the PSI model relatively large. Moreover, low *ρ*_*b*_ signifies a low amount of fuel and, by extension, a low amount of energy is generated from combustion (*H*_*comb*_). As such, natural peatlands have *H*_*comb*_/*H*_*ign*_ quotients of less than one (Fig. 4) explaining why most peatland fires are characterized by shallow burn depths (e.g. ref. 5).

When peatland drainage is performed to enhance tree growth (forestry), to permit the growth of crops (agriculture), or to permit the usage of heavy machinery to extract surface peat (mining), peatland WTs are substantially lowered. In our literature survey, we found that the average WT depth in drained peatlands was 41 cm which is 24 cm lower than in natural peatlands. The lowest WTs (60–80 cm) were found in dry boreal continental peatlands. A WT depth of 40–50 cm is considered optimal for tree production and is normally targeted when peatlands are drained for forestry^28^. A lower WT position enables the establishment of a dense tree cover^29^ (Fig. 5e) that increases both wildfire propagation^30^ and depth of burn^25^ through multiple positive ecohydrological feedbacks that lower peat *m* levels (e.g. WT-afforestation feedback, see ref. 2 for details). The expansion of trees into non-forested peatlands increases root uptake for transpiration and canopy interception (and concomitant evaporation), reducing the net input of water to the peatland and a lowering of the WT, that in turn promotes further afforestation. Moreover, this drying is enhanced by the loss of ground-layer *Sphagnum* mosses that occurs when shade-dwelling feather mosses (e.g. genera *Polytricum* and *Pleurozium*) outcompete *Sphagnum*. Declining *Sphagnum* moss cover reduces the ability of surface peat to retain moisture and increases the risk of desiccation in the top moss layer (Fig. 5e). Over time, feather mosses and vascular plants facilitate the formation of much denser peat (higher *ρ*_*b*_) due to their higher decay rates compared to most *Sphagnum* species (e.g. ref. 3). Bulk density is further increased following drainage due to increased peat compaction and accelerated peat decomposition (e.g. the WT-peat deformation feedback^2^). Briefly, this process takes place because as the WT declines effective stress increases and the resulting peat compression causes pore spaces to collapse, increasing peat *ρ*_*b*_. Nevertheless, as expected from these three processes, we found a clear difference in peat properties between drained and natural peatlands in both North America and Northern Europe (Fig. 2). Drained and natural peatlands have a similar *ρ*_*b*_ in deeper peat layers (below 40 cm), but in the top layers drained peatlands have a much higher *ρ*_*b*_. Mined peatlands have the highest *ρ*_*b*_ because of the surface moss and peat removed, exposing deeper and denser peat. High *ρ*_*b*_ is also associated with a decrease in specific yield (*S*_*y*_), (i.e. describes the amount of WT decline per unit of water loss) and, therefore, a similar water deficit will cause a greater WT decline in drained peatlands compared to natural peatlands. Hence, differences in peat properties lead to large differences in WT responses to water deficits, as observed in our WT response modelling (Fig. 3).

The decrease in surface *m* causes a decrease in *H*_*ign*_, while the increase in *ρ*_*b*_ increases *H*_*comb*_ resulting in an increase in *H*_*comb*_/*H*_*ign*_ quotients and an increase in the potential for deep burning^25^, which is supported by our burn severity modelling results. These results suggest a high risk of deep burning (up to 30 cm) for a WT of 40 cm and much deeper burns are possible at a WT of 80 cm. Given that a drained peatland has a WT depth of 20 cm at the start of the growing season (e.g. ref. 31), only a further decline of 20 cm is needed to reach a depth where deep burns are likely to occur. Such a WT decline corresponds to a water deficit of only 50 mm, which is highly likely to occur during drought^32^. Given the predicted increase in future water deficit of 50–100 mm for Europe, the risk of extreme WT drawdowns and catastrophic deep burning for European drained and mined peatlands will increase. However, several negative hydrological feedbacks (e.g. WT-moss surface resistance and albedo feedback, WT-transmissivity feedback, see ref. 2 for details) will likely mitigate natural peatlands from experiencing an increased risk of wildfire.

The drained and mined peatland burn severity modelling results fall within the depths of burn reported for drained peatlands 17.5–47 cm^10^,^14^,^33^, and the only mined peatland (24 cm^18^). As such, the effect of peatland management on wildfire carbon losses can be large. For example, the up to 30 cm deep burn (at WT = 40 cm) corresponds to a carbon loss of 21 kg m^−2^ (or circa 200 t C ha^−1^) assuming a *ρ*_*b*_ of 125 kg m^−3^ (Fig. 2) and 55% peat carbon content^34^. For the extreme 80 cm WT depth scenario the carbon losses would exceed 35–40 kg m^−2^, which is similar to the tropical peat fire values^13^. Deeper burns release old legacy carbon that has been locked in the peatland for centuries^10^. Using long-term carbon accumulation rates^1^ carbon losses of 21 and 35 kg m^−2^ through combustion is equivalent to over 600 and 1,000 years of carbon sequestration, respectively. Given the range of fire return interval for peatlands presented earlier the carbon lost in these wildfires will very likely not be balanced by between-fire carbon sequestration^9^. Moreover, given that recent research has demonstrated that deep burning of drained northern peatlands can likely convert a peat moss-dominated peatland to a non-carbon accumulating shrub-grass ecosystem with a low intensity, high frequency wildfire regime^35^, the further depletion of the legacy of stored peat carbon is highly probable.

### Peatland restoration mitigates peatland wildfire carbon losses

In order to shift a drained (and mined) peatland back to a system dominated by negative feedbacks^2^ that limit WT declines and maintain a high *m*, rewetting (Fig. 5f) and/or the establishment of a new *Sphagnum* peat moss layer is necessary (Fig. 5c,f). Rewetting of managed peatlands, i.e. restoring a WT closer to the surface (25 cm, Fig. 4), may not be sufficient to initiate the rapid recolonization of *Sphagnum* peat mosses^36^. The surface layer can be disconnected from the WT during drought leading to a drying of the peat^37^, thus making it hard for *Sphagnum* to establish and compete with feather mosses^38^. Increasing light availability via canopy removal^39^,^40^ (Fig. 5f) in forested drained peatlands can help *Sphagnum* to compete with feather mosses, but the effects of this practice alone may be marginal^40^,^41^. As such, the complete recovery in *Sphagnum* cover and species composition may require decades^31^,^39^,^42^. Without the recovery of a new moss layer rewetting alone is not sufficient to reduce the risk of deep burning (Fig. 4) unless the WT remains at the peat surface. In situations where permanently flooded conditions are not possible, active restoration with the spreading of mulch and *Sphagnum* fragments is necessary to achieve a wetter peat surface and a rapid *Sphagnum* establishment^36^,^38^ (Fig. 5c). Consequently, a combination of thinning, rewetting and restoration of a surface layer of growing *Sphagnum* (Fig. 5c,f) is necessary to rapidly decrease the potential of deep burns in managed peatlands (Fig. 4).

The restoration scenarios described in Fig. 5 focusses on *Sphagnum* dominated peatlands and does not include rich fens and blanket peatlands that are primarily comprised of brown mosses and sedges/graminoids, respectively. When subjected to drainage their characteristic flora is often lost and it can be a challenge to successfully restore these habitats. In blanket peatlands, a slow recovery of the hydrological function is observed after ditch blocking^43^, and the risk of deep burns in afforested peatlands may not be completely prevented by this action. For rich fens, restoration may be successful in terms of eliminating the potential of deep burns, because *Sphagnum* mosses are likely to invade after ditch-blocking, but the biodiversity goal can be missed unless additional treatments such as species introduction and top-soil removal are performed^40^. If a rich fen is successfully restored with brown mosses, it will likely be more vulnerable to deep burns compared to a *Sphagnum*-dominated scenario as brown mosses have lower moisture retention and capillary rise compared to *Sphagnum*^44^. However, because rich fen restoration aims at achieving a shallow WT, deep burns are unlikely in this habitat despite higher *ρ*_*b*_ in brown moss/herbaceous peat^27^.

### Peatland management in a changing climate

Our study explores the underlying processes responsible for the increased risk of deep burns in managed northern peatlands and provides a more comprehensive knowledge to assess and mitigate the risk of deep burns in drained peatlands. We demonstrate that the large area of managed peatlands in Europe will likely be more vulnerable to future wildfire due to climate change. Given the elevated risks of carbon losses, ecosystem shifts^35^ and health effects^19^ associated with wildfire in drained and mined peatlands, we support calls^24^,^45^ for large-scale restoration efforts on managed peatlands. While drained peatlands are a carbon source (2 t C ha^−1^ yr^−1 ^45) and restoration efforts often target the restoration of wetland habitat and the carbon sequestration function, we argue that the avoidance of catastrophic wildfires (>200 t C ha^−1^) should be a greater driver for improving peatland management practices.

## Methods

### Historical and future water deficit

We used data from the Canadian Regional Climate Model (CRCM 4^46^) with a horizontal resolution of 0.44° (~50 km) for North America (>42°N) and Europe that encompasses boreal and temperate biomes. Model outputs of daily simulated weather data were used to compute the average annual maximum water deficit for a historical (1981–2000) and future (2041–2060) period under the RCP 8.5 emission scenario^47^. The maximum annual water deficit was determined from the cumulative sum of precipitation (*P*) and potential evapotranspiration (*PET*), where *PET* was calculated according to a version of the Penman equation using the Shuttleworth^48^ estimate for open water aerodynamic resistance. Ground heat flux (*G*) was not included in the calculation of *PET*, since it was not an archived output variable for CRCM and, at a daily time step, *G* is a small component of the energy balance in peatlands^49^,^50^. Nevertheless, in order to capture the broad effects of *G* and bulk surface resistance on *ET*, we applied an empirical coefficient to our water deficit calculation. We calculated maximum annual water deficit as *P*–0.77*PET*. The empirical coefficient (0.77) was derived from archived Fluxnet data^51^, where average *ET*/*PET* was calculated from several years of eddy covariance data from Mer Bleue bog in south-east Ontario (bog) and Degerö Stormyr in Sweden (poor fen). Although not directly comparable with *ET*/*PET* a study^52^ report Priestley-Taylor *α* values of 0.62 and 0.75 for pristine bogs and fens, respectively, in Finland, and reference other studies which range between 0.51–0.98.

### Modelled water table position

In order to estimate the effect of peatland management on WT drawdown for a given water deficit, we collected data (new and literature values) on bulk density (*ρ*_*b*_) profiles in natural, drained, mined and restored peatlands from paired studies. These data cover a range of peatland types, from rich/intermediate fens to bogs. Data on *ρ*_*b*_ profiles in boreal peatlands, representing the Euro-Asia continent, were taken from a countrywide study that measured *ρ*_*b*_ in drained (N = 651) and natural (N = 360) peatlands at a large spatial scale^34^. Data from a mined peatland was extracted from a separate study^53^. For Canada, we extracted data from several studies^2^,^54^,^55^,^56^, and also collected data at two new sites. At the new sites (Wainfleet, Ontario and Maclennan, Alberta), 12 cores (10 cm diameter) were collected encompassing microtopographic (hummock-lawns) and spatial variation. To avoid compaction of the samples we cut out the cores carefully and froze them before they were cut into 5 cm sections. Samples were thereafter dried and weighed. To evaluate how representative the natural peatlands are at a larger scale, we compared the *ρ*_*b*_ profiles with profiles reported in a peat properties database for northern peatlands^27^. We removed permafrost and high altitude (>1000 m) sites because peatland drainage is generally not practiced in these regions. From the public database, we used 28 *ρ*_*b*_ profiles from sites meeting the above criteria (25 of 70 sites).

In order to represent natural, drained, mined, and restored peatland sites, we generated *ρ*_*b*_ profiles by using the aforementioned near-surface *ρ*_*b*_ data (0–50 cm) and thereafter, based on the selected *ρ*_*b*_ profiles from^27^, applied a linear relationship between *ρ*_*b*_ and peat depth down to 1.5 m, where we prescribed a value of 150 kg m^−3^. For the restored scenarios, we assumed that site restoration was achieved by re-establishing a shallow WT and the re-generation of *Sphagnum* cover. We prescribed a 12 and 28 cm thick restored layer, with the same upper *ρ*_*b*_ profile as the natural site, as these new moss/peat thicknesses have been shown to maintain a relatively shallow WT for a range of maximum summer water deficits of 40 to 100 mm at mined peatland in Quebec, Canada^42^. Below the 12 and 28 cm restored layer, we used the *ρ*_*b*_ profiles from the mined and drained peatlands. In order to estimate WT response to water deficit, the depth-dependence of specific yield (*S*_*y*_) needs to be known because *S*_*y*_ is the depth of water that must be added to or removed from a soil column in order to cause a unit rise or fall in the WT. We estimated *S*_*y*_ from the *ρ*_*b*_ data using the equation from Duke (1972), where *ρ*_*b*_ dependent moisture retention parameters were derived for *Sphagnum* peat^57^. Water table position was then calculated by iteratively solving for the WT depth where the water deficit equalled the depth integrated *S*_*y*_ values. For comparative purposes, we used water deficits at a continental and maritime location in both North America and Europe where managed peatlands are common. Additional lateral water losses due to drainage were not considered, thus our modelled WT values are a conservative estimate of WT drawdown given our simple treatment of ET.

### Peat burn severity modelling

A literature review of managed peatlands informed our peat burn severity modelling scenarios. WT depths for drained peatlands were conservatively based on data from the mid-points between drainage ditches. Estimated average WT depths (growing season) in peatlands drained for forestry and natural peatlands were 41 cm (sd = 18, n = 37, Supplementary Table 2) and 17 cm (sd = 10, n = 37, Supplementary Table 2), respectively. A few studies reported an average WT depth close to 80 cm in peatlands drained for forestry, which were generally from dry, continental peatlands (e.g. Alberta, Canada). We also observed that the average WT in mined peatlands (active or abandoned) is not frequently reported, but data suggest a WT depth of 50–100 cm (Supplementary Table 2). As such, peat burn severity was modelled at three WT depths: (i) 40 cm which is a typical WT in drained peatlands, (ii) 80 cm which is a more extreme scenario supported by the difference in WT decline in drained peatlands between wetter and drier climatic zones, and (iii) 25 cm which represents a rewetting scenario close to natural conditions.

Peat burn severity (depth of burn) was estimated using the Peat Smouldering and Ignition model^25^,^26^. This simple modelling approach is suitable because landscape patterns of smouldering are almost exclusively dictated by the hydrophysical properties (i.e. *ρ*_*b*_ and gravimetric moisture content, *m*) of the peat, and the model is supported by detailed physics-based models^58^,^59^. Values of *m* throughout the peat profiles were estimated using peat moisture retention curves as a function of *ρ*_*b*_^57^, exponentially declining soil tension profiles^60^, and fixed WT depths of 25, 40 and 80 cm (see above). Peat burn severity was then estimated by the quotient between energy released via combustion (*H*_*comb*_) and energy required for ignition (*H*_*ign*_) between successive peat layers (5 cm thick in this study) in a peat profile (see^25^,^60^ for full model description). A *H*_*comb*_/*H*_*ign*_ quotient <1 between two layers indicates a low smouldering potential because the energy sourced from combusting peat does not exceed the energy required for the subsequent combustion of the underlying peat. Consequently, vertical smouldering propagation will cease at a quotient <1 and continue at a quotient >1. However, this assumes that the downward efficiency of the energy released is 100% but previously reported downward efficiencies range from 30–90%^61^,^62^. A *H*_*comb*_/*H*_*ign*_ quotient of two, for example, indicates that only 50% of the energy produced by the combusting peat layer would need to be transferred downward to the underlying layer in order for smouldering to propagate. Thus, a more realistic smouldering propagation cut-off is likely between 1–1.5.

## Additional Information

**How to cite this article**: Granath, G. *et al*. Mitigating wildfire carbon loss in managed northern peatlands through restoration. *Sci. Rep.* **6**, 28498; doi: 10.1038/srep28498 (2016).

## Supplementary Material

Supplementary Information

## References

[b1] Yu Z. C. Northern peatland carbon stocks and dynamics: a review. Biogeosciences 9, 4071–4085 (2012).

[b2] Waddington J. M. . Hydrological feedbacks in northern peatlands. Ecohydrology 8, 113–127 (2015).

[b3] Moore T. R., Bubier J. L. & Bledzki L. Litter Decomposition in temperate peatland ecosystems: The effect of substrate and site. Ecosystems 10, 949–963 (2007).

[b4] Rydin H. & Jeglum J. K. The Biology of Peatlands, 2nd edn (Oxford University Press Inc., New York, 2013).

[b5] Shetler G., Turetsky M. R., Kane E. & Kasischke E. *Sphagnum* mosses limit total carbon consumption during fire in Alaskan black spruce forests. Canadian Journal of Forest Research 38, 2328–2336 (2008).

[b6] Turetsky M., Wieder K., Halsey L. & Vitt D. Current disturbance and the diminishing peatland carbon sink. Geophysical Research Letters 29, 21.1–21.4 (2002).

[b7] Benscoter B. W. & Wieder R. K. Variability in organic matter lost by combustion in a boreal bog during the 2001 Chisholm fire. Canadian Journal of Forest Research 33, 2509–2513 (2003).

[b8] Pitkänen A., Turunen J. & Tolonen K. The role of fire in the carbon dynamics of a mire, eastern Finland. The Holocene 9, 453–462 (1999).

[b9] Wieder R. K. . Postfire carbon balance in boreal bogs of Alberta, Canada. Global Change Biology 15, 63–81 (2009).

[b10] Turetsky M. R., Donahue W. F. & Benscoter B. W. Experimental drying intensifies burning and carbon losses in a northern peatland. Nature Communications 2, 514 (2011). 10.1038/ncomms1523 22044993

[b11] Lavoie C. & Pellerin S. Fires in temperate peatlands (southern Quebec): past and recent trends. Canadian Journal of Botany 85, 263–272 (2007).

[b12] Magnan G., Lavoie M. & Payette S. Impact of fire on long-term vegetation dynamics of ombrotrophic peatlands in northwestern Québec, Canada. Quaternary Research 77, 110–121 (2012).

[b13] Page S. E. . The amount of carbon released from peat and forest fires in Indonesia during 1997. Nature 420, 61–65 (2002). 12422213 10.1038/nature01131

[b14] Reddy A. D. . Quantifying soil carbon loss and uncertainty from a peatland wildfire using multi-temporal LiDAR. Remote Sensing of Environment 170, 306–316 (2015).

[b15] van der Werf G. R. . Global fire emissions and the contribution of deforestation, savanna, forest, agricultural, and peat fires (1997–2009). Atmos. Chem. Phys. 10, 11707–11735 (2010).

[b16] Yule C. M. Loss of biodiversity and ecosystem functioning in Indo-Malayan peat swamp forests. Biodiversity and Conservation 19, 393–409 (2008).

[b17] Sahani M. . A case-crossover analysis of forest fire haze events and mortality in Malaysia. Atmospheric Environment 96, 257–265 (2014).

[b18] MacDougall H. Peat burn severity in a drained and mined peatland. Honours B.Sc., McMaster University, Hamilton, Canada (2014).

[b19] Shaposhnikov D. . Mortality related to air pollution with the Moscow heat wave and wildfire of 2010. Epidemiology (Cambridge, Mass.) 25, 359–364 (2014). 10.1097/EDE.0000000000000090 PMC3984022 24598414

[b20] Flannigan M., Stocks B., Turetsky M. & Wotton M. Impacts of climate change on fire activity and fire management in the circumboreal forest. Global Change Biology 15, 549–560 (2009).

[b21] Turetsky M. R., Amiro B. D., Bosch E. & Bhatti J. S. Historical burn area in western Canadian peatlands and its relationship to fire weather indices. Global Biogeochemical Cycles 18, GB4014 (2004).

[b22] Joosten H. IMCG Global Peatland Database. *Technical Report.* Available at: http://www.imcg.net/pages/publications/imcg-materials.php. (Date of access: 01/10/2015) (2004).

[b23] Paustian K. . Climate-smart soils. Nature 532, 49–57 (2016). 27078564 10.1038/nature17174

[b24] Wet, wet, wet. *The Economist* (2010, Dec 16).

[b25] Benscoter B. W. . Interactive effects of vegetation, soil moisture and bulk density on depth of burning of thick organic soils. International Journal of Wildland Fire 20, 418–429 (2011).

[b26] Thompson D. K., Wotton B. M. & Waddington J. M. Estimating the heat transfer to an organic soil surface during crown fire. International Journal of Wildland Fire 24, 120 (2015).

[b27] Loisel J. . A database and synthesis of northern peatland soil properties and Holocene carbon and nitrogen accumulation. The Holocene 24, 1028–1042 (2014).

[b28] Paavilainen E. & Päivänen J. Peatland forestry. Ecology and principles. Ecological Studies 111 (Springer-Verlag, Berlin, 1995).

[b29] Lieffers V. J. & Rothwell R. L. Rooting of peatland black spruce and tamarack in relation to depth of water table. Canadian Journal of Botany 65, 817–821 (1987).

[b30] Johnston D., Turetsky M., Benscoter B. & Wotton B. Fuel load, structure, and potential fire behaviour in black spruce bogs. Canadian Journal of Forest Research 45, 888–899 (2015).

[b31] Komulainen V.-M., Tuittila E.-S., Vasander H. & Laine J. Restoration of drained peatlands in southern Finland: initial effects on vegetation change and CO_2_ balance. Journal of Applied Ecology 36, 634–648 (1999).

[b32] Waddington J. . Examining the utility of the Canadian Forest Fire Weather Index System in boreal peatlands. Canadian Journal of Forest Research 42, 47–58 (2011).

[b33] Davies G. M., Gray A., Rein G. & Legg C. J. Peat consumption and carbon loss due to smouldering wildfire in a temperate peatland. Forest Ecology and Management 308, 169–177 (2013).

[b34] Minkkinen K. & Laine J. Effect of forest drainage on the peat bulk density of pine mires in Finland. Canadian Journal of Forest Research 28, 178–186 (1998).

[b35] Kettridge N. . Moderate drop in water table increases peatland vulnerability to post-fire regime shift. Scientific Reports 5, 8063 (2015). 25623290 10.1038/srep08063 PMC4306970

[b36] Rochefort L., Quinty F., Campeau S., Johnson K. & Malterer T. North American approach to the restoration of *Sphagnum* dominated peatlands. Wetlands Ecology and Management 11, 3–20 (2003).

[b37] McCarter C. P. & Price J. The hydrology of the BoisdesBel peatland restoration: hydrophysical properties limiting connectivity between regenerated Sphagnum and remnant vacuum harvested peat deposit. Ecohydrology 8, 173–187 (2015).

[b38] Price J. Soil moisture, water tension, and water table relationships in a managed cutover bog. Journal of Hydrology 202, 21–32 (1997).

[b39] Haapalehto T. O., Vasander H., Jauhiainen S., Tahvanainen T. & Kotiaho J. S. The effects of peatland restoration on water-table depth, elemental concentrations, and vegetation: 10 years of changes. Restoration Ecology 19, 587–598 (2011).

[b40] Hedberg P. . Vegetation recovery after multiple-site experimental fen restorations. Biological Conservation 147, 60–67 (2012).

[b41] Kettridge N. . The ecohydrology of forested peatlands: Simulating the effects of tree shading on moss evaporation and species composition. Journal of Geophysical Research: Biogeosciences 118, 422–435 (2013).

[b42] Lucchese M. . Organic matter accumulation in a restored peatland: Evaluating restoration success. Ecological Engineering 36, 482–488 (2010).

[b43] Holden J., Wallage Z. E., Lane S. N. & McDonald A. T. Water table dynamics in undisturbed, drained and restored blanket peat. Journal of Hydrology 402, 103–114 (2011).

[b44] Goetz J. D. & Price J. S. Ecohydrological controls on water distribution and productivity of moss communities in western boreal peatlands, Canada. Ecohydrology 9, 138–152 (2016).

[b45] Tanneberger F. & Wichtmann W. (eds.) Carbon credits from peatland rewetting: climate–biodiversity–land use; science, policy, implementation and recommendations of a pilot project in Belarus (Schweizerbart, Stuttgart, 2011).

[b46] Music B. & Caya D. Evaluation of the hydrological cycle over the Mississippi River basin as simulated by the Canadian Regional Climate Model (CRCM). Journal of Hydrometeorology 8, 969–988 (2007).

[b47] Collins M. . Long-term climate change: projections, commitments and irreversibility. Pages 1029 to 1076. In Intergovernmental Panel on Climate Change (ed.) Climate Change 2013–The Physical Science Basis, 1029–1136 (Cambridge University Press, Cambridge, http://ebooks.cambridge.org/ref/id/CBO9781107415324A032 2014). (Date of access: 01/05/2016).

[b48] Shuttleworth W. J. Evaporation. In Maidment D. R. (ed.) Handbook of hydrology (McGraw-Hill, United States, 1993).

[b49] Admiral S. W., Lafleur P. M. & Roulet N. T. Controls on latent heat flux and energy partitioning at a peat bog in eastern Canada. Agricultural and Forest Meteorology 140, 308–321 (2006).

[b50] Moore P. A., Pypker T. G. & Waddington J. M. Effect of long-term water table manipulation on peatland evapotranspiration. Agricultural and Forest Meteorology 178–179, 106–119 (2013).

[b51] Baldocchi D. . FLUXNET: A new tool to study the temporal and spatial variability of ecosystem–scale carbon dioxide, water vapor, and energy flux densities. Bulletin of the American Meteorological Society 82, 2415–2434 (2001).

[b52] Gong J. . Modeling water table changes in boreal peatlands of Finland under changing climate conditions. Ecological modelling 244, 65–78 (2012).

[b53] Wilson D. . Rewetting of cutaway peatlands: are we re-creating hot spots of methane emissions? Restoration Ecology 17, 796–806 (2009).

[b54] Shantz M. A. & Price J. S. Hydrological changes following restoration of the Bois-des-Bel Peatland, Quebec, 1999–2002. Journal of Hydrology 331, 543–553 (2006).

[b55] Ketcheson S. J. & Price J. S. Characterization of the fluxes and stores of water within newly formed *Sphagnum* moss cushions and their environment. Ecohydrology 7, 771–782 (2014).

[b56] Taylor N. & Price J. Soil water dynamics and hydrophysical properties of regenerating *Sphagnum* layers in a cutover peatland. Hydrological Processes 29, 3878–3892 (2015).

[b57] Moore P., Morris P. & Waddington J. Multi-decadal water table manipulation alters peatland hydraulic structure and moisture retention. Hydrological Processes 29, 2970–2982 (2015).

[b58] Huang X., Rein G. & Chen H. Computational smoldering combustion: Predicting the roles of moisture and inert contents in peat wildfires. Proceedings of the Combustion Institute 35, 2673–2681 (2015).

[b59] Prat-Guitart N., Rein G., Hadden R. M., Belcher C. M. & Yearsley J. M. Effects of spatial heterogeneity in moisture content on the horizontal spread of peat fires. Science of The Total Environment, 10.1016/j.scitotenv.2016.02.145 (2016). 27000715

[b60] Lukenbach M. C. . Hydrological controls on deep burning in a northern forested peatland. Hydrological Processes 29, 4114–4124 (2015).

[b61] Schneller M. & Frandsen W. A stirred water calorimeter for measuring heat flux from smoldering combustion. International Journal of Wildland Fire 8, 129–135 (1998).

[b62] Frandsen W. Heat flow measurements from smoldering porous fuel. International Journal of Wildland Fire 8, 137–145 (1998).

