# Supplementary material for: Mitigating wildfire carbon loss in managed northern peatlands through restoration
Source: Sci Rep. 2016 Jun 27;6:28498. doi: 10.1038/srep28498 (PMC4921962; doi:10.1038/srep28498)
Supplement: Supplementary Information [file srep28498-s1.pdf]

Supplementary Information

MITIGATING WILDFIRE CARBON LOSS IN MANAGED NORTHERN PEATLANDS THROUGH RESTORATION

G. GRANATH, P.A. MOORE, M.C. LUKENBACH, J.M. WADDINGTON

CONTENT:

Figure S1: Modelled risk of deep burning and mean depth of burn in two burned peatlands.
Table S1: Area drained peatland per country.
Table S2: WT depths in drained, mined and natural peatlands.

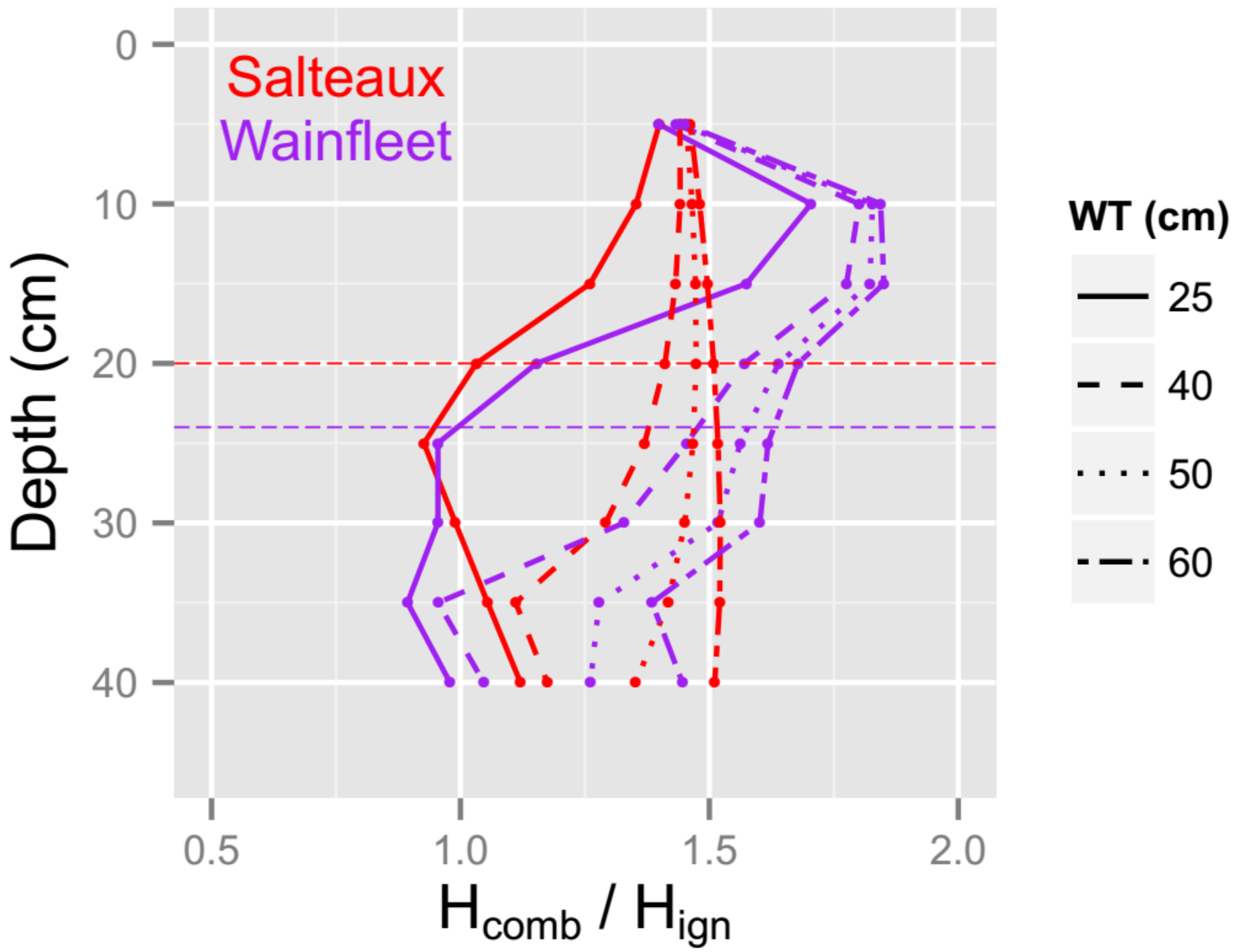

Figure 1S. Variability in combustion risk based on likely WT positions during the wildfire of Salteaux (drained for forestry), AB, and Wainfleet (mined and abandoned), ON, Canada. The horizontal dashed lines represent the mean depth of burn (DOB) for each site. This figure suggests that the water table during the fires was below 40 cm. An H_{comb}/H_{ign} quotient <1 indicates that the combustion risk is zero (*i.e.* downward smouldering propagation ceases), but a more realistic cut-off is between 1-1.5.

Table S1. Top 15 Northern hemisphere countries with the largest peatland area (Mha) drained for forestry and peat mining (large scale peat removal for horticulture or fuel). Data from the IMCG data base.

**Country**	**Forestry Drainage (Mha)**	**Mining Drainage (Mha)**	**Drainage Total (Mha)**
1. Russia (Europe)	2.90	8.80	11.7
2. Finland	5.94	0.08	6.02
3. Sweden	1.00	0.01	1.01
4. Norway	0.41	0.03	0.44
5. Belarus	0.38	0.04	0.42
6. Poland	0.24	0.08	0.32
7. United Kingdom	0.22	0.01	0.23
8. Russia (Asia)	0.10	0.10	0.20
9. Estonia	0.16	0.03	0.19
10.Ukraine	0.15	0.01	0.16
11. Lithuania	0.13	0.01	0.14
12. Ireland	0.01	0.11	0.12
13. Latvia	0.07	0.04	0.11
14. Denmark	0.07	0.00	0.07
15. Canada	0.02	0.02	0.04

Table S2. Published data on average water table (WT) depth over the growing season for drained and mined boreal/temperate peatlands, and for natural (undrained) reference sites when reported.

Peatland type	Mean WT drained (cm)	Mean WT natural (cm)	Location	Study
fen	31	4	Finland	Regina et al. 1996
fen	38	21	Finland	Regina et al. 1996
fen	50		Finland	Regina et al. 1996
fen		5	Finland	Regina et al. 1996
bog	36	28	Finland	Regina et al. 1996
bog	24	18	Finland	Regina et al. 1996
bog	13	11	Finland	Regina et al. 1996
bog	20	16	Finland	Regina et al. 1996
bog	27	12	Finland	Regina et al. 1996
bog		13	Finland	Regina et al. 1996
bog	20	15	Finland	Alm et al. 1999
bog	21	16	Finland	Alm et al. 1999
bog		17	Finland	Alm et al. 1999
fen	30	2	Finland	Alm et al. 1999
fen	38	5, 10	Finland	Alm et al. 1999
fen	40		Finland	Alm et al. 1999
fen	46		Finland	Komulainen et al. 1999
bog	35		Finland	Komulainen et al. 1999
fen	33	4	Finland	Laine et al. 1996
fen	33	10	Finland	Laine et al. 1996
bog	31	19	Finland	Laine et al. 1996
bog	24	11	Finland	Laine et al. 1996
fen	75	30	Canada, AB	Silins & Rothwell 1999
fen	80	35	Canada, AB	Silins & Rothwell 1999
fen	85	22	Canada, AB	Hillman 1992
fen	38	35	Canada, ON	Berry & Jeglum 1991
fen	41	32	Canada, ON	Berry & Jeglum 1991
fen	72	22	Canada, ON	Berry & Jeglum 1991
fen	38	26	Canada, ON	Berry & Jeglum 1991
bog	28	22	Canada, QB	Prevost et al. 1997
bog	43		Sweden*	Heikurainen 1973
bog	30		Polen	Trettin et al. 1996
bog	50		W Russia	Trettin et al. 1996
bog	55		W Russia	Trettin et al. 1996
bog	50		Belarus	Trettin et al. 1996
bog	75		Belarus	Trettin et al. 1996
bog	45		Estonia	Trettin et al. 1996
Mean	41	17		
mined	63		Finland	Regina et al. 1996
mined	73		Finland	Regina et al. 1996

*Mean of 5 sites spread over Sweden.

References TABLE S2

Alm, J., Saarnio, S., Nykanen, H., Silvola, J., & Martikainen, P. 1999. Winter CO2, CH4 and N2O fluxes on some natural and drained boreal peatlands. Biogeochemistry. 44: 163-186.

Berry, G. J., & Jeglum, J. K. 1991. Hydrology of drained and undrained black spruce peatlands: groundwater table profiles and fluctuations. Canada-Ontario Forest Resource Development Agreement.

Heikurainen, L. 1973. Skogsdikning. Norsteds & Söners förlag, Stockholm, 444 pp. (In Swedish)

Hillman, G. R. 1992. Some hydrological effects of peatland drainage in Alberta's boreal forest. Canadian Journal of Forest Research. 22: 1588-1596.

Komulainen, V.-M., Tuittila, E.-S., Vasander, H. and Laine, J. 1999. Restoration of drained peatlands in southern Finland: initial effects on vegetation change and CO2 balance. Journal of Applied Ecology. 36: 634–648.

Laine, J., Silvola, J., Tolonen, K., Alm, J., Nykänen, H., Vasander, H., Sallantaus, T., Savolainen, I., Sinisalo, J., & Martikainen, P. J. 1996. Effect of water-level drawdown on global climatic warming: Northern peatlands. Ambio. 25: 179-184.

Prevost, M., Belleau, P., & Plamondon, A. P. 1997. Substrate conditions in a treed peatland: responses to drainage. Ecoscience. 4:543-554

Regina, K., Nykanen, H., Silvola, J., & Martikainen, P. J. 1996. Fluxes of nitrous oxide from boreal peatlands as affected by peatland type, water table level and nitrification capacity. Biogeochemistry. 35: 401-418.

Silins, U., & Rothwell, R. L. 1999. Spatial patterns of aerobic limit depth and oxygen diffusion rate at two peatlands drained for forestry in Alberta. Canadian Journal of Forest Research. 29: 53-61.

Trettin, C. C., Jurgensen, M. F., Grigal, D. F., Gale, M. R., & Jeglum, J. R. 1996. Northern forested wetlands ecology and management. CRC Press.
